# Activity in nature mediates a park prescription intervention’s effects on physical activity, park use and quality of life: a mixed-methods process evaluation

**DOI:** 10.1186/s12889-021-10177-1

**Published:** 2021-01-22

**Authors:** Nicholas Petrunoff, Jiali Yao, Angelia Sia, Alwyn Ng, Anbumalar Ramiah, Michael Wong, Jane Han, Bee Choo Tai, Léonie Uijtdewilligen, Falk Müller-Riemenschneider

**Affiliations:** 1 Saw Swee Hock School of Public Health, National University of Singapore, Block MD1, 12 Science Drive 2, #10-01, Singapore, 117549 Singapore; 2 Centre for Urban Greenery & Ecology, National Parks Board Singapore, 1E Cluny Rd., Singapore Botanic Gardens, Singapore, 259569 Singapore; 3 Health for Life Centre, Khoo Teck Puat Hospital, Alexandra Health Pte Ltd. 90 Yishun Central, Singapore, 768828 Singapore; 4 Institute for Social Medicine, Epidemiology and Health Economics, Charite University Medical Centre Berlin, Luisenstrasse 57, 10117 Berlin, Germany

**Keywords:** Parks, Urban green space, Physical activity, Process evaluation, Mediation analysis

## Abstract

**Background:**

This process evaluation explored the implementation and mechanisms of impact of a Park Prescription Intervention trial (PPI), including the effects of hypothesised mediators (motivation, social support, recreational physical activity [PA], park use and park PA) on trial outcomes.

**Methods:**

Participants from the community were randomly allocated to intervention (*n* = 80) or control (n = 80) group. The intervention included baseline counselling, a prescription of exercise in parks, materials, three-month follow-up counselling and 26 weekly group exercise sessions in parks. Process evaluation indicators were assessed at three- and six-months. Implementation indicators included participation rates in intervention components and survey questions plus focus group discussions (FGDs) to understand which components participants valued. FGDs further assessed barriers and facilitators to intervention participation. To explore mechanisms of impact, linear regression was used to compare objectively measured PA between quantiles of group exercise participation. Structural equation modelling (SEM) explored hypothesised mediation of the significant intervention effects. Framework analysis was conducted for FGDs.

**Results:**

Participants were middle-aged (mean 51, SD ± 6.3 years), predominantly female (79%) and of Chinese ethnicity (81%). All intervention participants received baseline counselling, the park prescription and materials, whilst 94% received the follow-up counselling. Mean minutes of moderate-to-vigorous PA/week (95% CI) differed by group exercise participation (*p* = 0.018): 0% participation (*n* = 18) 128.3 (69.3, 187.2) minutes, > 0–35.9% participation (n = 18) 100.3 (36.9, 163.6) minutes, > 35.9–67.9% participation (*n* = 17) 50.5 (− 4.9, 105.9) minutes and > 67.9% participation (n = 18) 177.4 (122.0, 232.8) minutes. Park PA at three-months had significant mediating effects (95% CI) on recreational PA 26.50 (6.65, 49.37) minutes/week, park use 185.38 (45.40, 353.74) minutes/month, park PA/month 165.48 (33.14, 334.16) minutes and psychological quality of life score 1.25 (0.19, 2.69) at six-months. Prioritising time with family and preferences for unstructured activities were barriers to intervention participation. Human interaction via follow-up or group exercise were facilitators.

**Conclusion:**

This process evaluation showed park PA consistently mediated effects of the PPI, suggesting activity in parks was a mechanism of its effects. To optimise effectiveness, participants’ preference for prioritising time with family through family involvement and tailoring the intervention to participants’ preferences for structured or unstructured PA could be considered in future studies.

**Trial registration:**

ClinicalTrials.gov NCT02615392, 26 November 2015.

**Supplementary Information:**

The online version contains supplementary material available at 10.1186/s12889-021-10177-1.

## Background

Physical inactivity is associated with a number of non-communicable diseases and premature mortality [1, 2]. According to global surveillance, about 25% of adults globally and 15% in South-East Asia reported insufficient physical activity (PA) [3]. A review including seven national studies that measured PA objectively with accelerometers estimated the prevalence of physical inactivity to be much higher (48–99%) [3, 4]. Therefore, systematic approaches which include effective interventions to address this global pandemic of physical inactivity are needed [5].

In 2018 the World Health Organisation set the target for a 15% relative reduction in the global prevalence of physical inactivity in adults by the year 2030 in the Global Action Plan on Physical Activity [6]. The action plan acknowledges the importance of parks as settings to engage in PA by stating ‘Enhance provision of, and opportunities for, more PA programmes and promotion in parks and other natural environments...’ The Park Prescription concept, which emerged from collaboration between the U.S. Centers for Disease Control and Prevention and the National Recreation and Parks Association, could represent one strategy to achieve these goals. In 2013 ‘Park Prescriptions’ were defined as ‘Programs designed in collaboration with healthcare providers. .. to utilise parks, trails and open space for improving. .. community health’ [7]. This is closely related to the concept of exercise prescription for people who are physically inactive [8]. Previous studies which examined prescription of PA in primary care settings showed that brief written or oral advice prescribing frequency, intensity, time and type of PA for inactive patients could increase their PA levels [9, 10]. Park Prescriptions specify exercise in parks since exposure to nature may confer additional benefits to physical and mental health beyond exercise alone [11], although the mechanisms are not fully understood [12–19].

The Park Prescription intervention trial (PPI), conducted in Singapore, tested the effect of an innovative program promoting PA in parks and found it was effective for improving park use, park PA, recreational PA and psychological quality of life (QoL) [20, 21]. Intervention process evaluations can help to understand which components worked, which didn’t and why by exploring the mechanisms of impact of the intervention on outcomes [22]. Although studies prescribing park PA have been published [11, 23–26], to our knowledge no process evaluations to understand the mechanisms of impact of such interventions exist in peer-reviewed scientific journals. The Medical Research Council guidance on process evaluation [22, 27] outlines three functions to be described – implementation (process, reach, dose, satisfaction, fidelity); mechanisms of impact (participants’ responses to and interaction with the intervention, mediators, unexpected pathways); and, context. To address this gap in the evidence, we conducted a mixed-methods process evaluation of the PPI following the Medical Research Council guidance.

This process evaluation complements the PPI by providing information on implementation dose and satisfaction as well as assessments of mechanisms of impact. This information could be used to adapt the original PPI to improve its effectiveness and to help us understand how interventions promoting PA in parks may improve health. It can also help to explain the PPI’s attainment and non-attainment of statistically significant outcomes [20]. For the current study, we aimed to answer four research questions. Firstly, to what extent do participants value the intervention and each of its components? Secondly, what do participants perceive as barriers and facilitators to participation in the group exercise component? Thirdly, do participants with a higher participation in the group exercise achieve greater objectively measured moderate-to-vigorous PA at six-month follow-up? Fourthly, do hypothesised factors mediate the effect of the intervention on the outcomes?

## Methods

### Study design and participants

The PPI was a two-arm randomised-controlled trial (RCT) that recruited Singaporeans aged 40–65 between April and December 2016 during freely available community-based health screenings in the northern part of Singapore. Participants satisfied several inclusion criteria [21], including no prior medical conditions preventing engagement in PA. Following baseline assessments, 160 participants were randomised in a 1:1 ratio into the intervention or the control group [20]. This trial was approved by the National Healthcare Group Domain Specific Review Board (DSRB) in Singapore [2015/00611-Park Prescription Trial]. Written informed consent was obtained from each participant for the collection and use of the data in future publication.

The process evaluation consisted of a mixed-methods study with an explanatory sequential design [28]. The qualitative focus group data was collected primarily to explain the quantitative results after the intervention was completed.

### Intervention components of the evaluated trial

Participants in the intervention group received face-to-face counselling on PA, during which they also completed a park prescription sheet with a trained study team member. The prescription sheet outlined a goal they committed to specifying the frequency, intensity, time and location of exercise in parks. Participants subsequently received a sheet to plan their weekly park PA and information brochures about parks in their neighbourhood. The first brochure was one developed for the trial, providing specific information on parks in the northern part of Singapore (within communities where participants resided) and their features, including walking trails, their difficulty level and locations of fitness corners. The second was an existing brochure produced by the National Parks Board, Singapore, containing a map and information on the Northern Explorer Loop (a series of parks in Singapore’s north connected by a network of walking and cycling paths). On the planning sheet, participants filled in the types of activities they aimed to do each week throughout the trial. Half-way through the trial, a trained study team member provided a brief counselling phone call, which assessed participants’ progress towards their set goals and included modification of those goals if necessary. In addition, participants were invited to join in a weekly one-hour outdoor structured and supervised PA program in the park for the entire intervention period of 6 months. Each one-hour session comprised moderate intensity aerobic activity and strength and balance exercises. To provide options in timing, two sessions of the structured PA program were organised in selected public parks located in the participants’ neighbourhood each week, one on a weekday evening and the other on Sunday mornings. The sessions utilised different areas and features of the parks, including walking trails and open spaces, to maximise participants’ exposure to greenery. To encourage attendance, participants received text message reminders prior to each weekly exercise session.

Participants in the control group continued with their daily routine. They received standard PA promotion materials that were not related to exercise in parks, which were existing publications by the Health Promotion Board, Singapore. In addition, they received all the information materials after the intervention group completed the study and they were also invited to join ongoing exercise classes upon study completion.

### Data collection and measures

Table 1 shows the baseline (T0), three-month follow-up (T1) and six-month follow-up (T2) measurements of this study which related to the implementation and mechanism of impact functions of the process evaluation. The measures for each function, time points, source and instruments are described further in this section, a brief description of the evaluation functions and components is provided in the table footnotes and a full description is provided elsewhere [21].

**Table 1:** Process evaluation function, components, data sources, time points and measures

Evaluation function^**a**^	Evaluation component	Data collection instruments	Data source	Time point	Measures
**Implementation**	Dose	Participation record	Participants – I^**b**^	Throughout six-month intervention from T0-T2	Proportion of each element of the intervention participated in
	Quality and Satisfaction	Surveys	Participants – I	T1 and T2	Satisfaction with intervention elements
		Focus groups.	Participants – I	Intervention completion	Which components participants valued
**Mechanism of impact**	Participant responses to the intervention	Surveys	Participants – I and C^**c**^	T1 and T2	Quality of life Recreational MVPA Park time in last month Park PA time
		Accelerometer	Participants – I and C	T2	MVPA Total PA
		Focus groups	Participants – I	Intervention completion	Explore reasons for participation
	Mediators	Surveys	Participants – I and C	T1 and T2	Motivation Social support for PA Park PA time Park time in last month Recreational MVPA
	Unexpected pathways and consequences	Surveys	Participants – I and C	T1 and T2	Motivation Park PA time Recreational MVPA
		Accelerometer	Participants – I	T2	MVPA Total PA
		Focus groups	Participants – I	Intervention completion	Barriers and facilitators to participation

^a^The evaluation function of implementation includes components to evaluate how the intervention was delivered, which include dose (i.e. the amount of each intervention component received), perceived quality of and satisfaction with the intervention components; mechanism of impact refers to evaluating why the intervention achieved (or did not achieve) its outcomes by exploring potential mediation effects through mediation analysis and focus group discussions to explore reasons for the results from the participants’ perspectives. I denotes the intervention group^**b**^ and C denotes the control group^**c**^. T0 = Baseline, T1 = three months, T2 = six months. MVPA = moderate-to-vigorous physical activity

#### Implementation measures

Measures for implementation dose included intervention group participation rates collected via participation records for the five intervention components. Participation in the initial counselling including the actual park prescription and providing program materials (planning sheet and brochures on local parks) was recorded at T0. Participation in follow-up counselling was recorded at T1 and participation in the group exercise was recorded throughout the 26-week intervention. The intervention group rated their satisfaction level and the quality of the PPI elements via two follow-up surveys (at T1, T2). In the survey at T1, participants were asked to rate the prescription, the prescriber and program materials. The survey at T2 included questions on quality of the phone follow-up counselling and satisfaction with the intervention overall. Focus groups, conducted after the intervention was completed (i.e. after T2), explored whether participants valued the intervention and each of its components and included in-depth discussions on the barriers and facilitators to participation.

#### Measures to assess the intervention’s mechanisms of impact on outcomes

The intervention’s mechanisms of impact were explored via survey measures administered at T1 and T2 amongst the intervention and control groups; the accelerometer at T2; and, via focus groups with intervention participants upon completion of the intervention. Group exercise participation measured each week over the 26-week intervention divided intervention participants into four subgroups: 0% participation (*n* = 18), > 0–35.9% participation (n = 18), > 35.9–67.9% participation (*n* = 17) and > 67.9% participation (n = 18,). Subgroup analyses explored whether levels of group exercise participation were related to MVPA time in minutes per week at T2 in the intervention group. This was measured over seven days, with three valid days being required as a minimum for data to be included in the analysis. Focus groups conducted at completion of the intervention explored whether the experiences of participants in the intervention group differed by level of participation.

The four outcomes measured by the self-report survey which improved significantly in the PPI intervention group compared to the control group at T2 were included in the mediation analysis: psychological quality of life (Domain 2 of WHO QoL BREF) [29], recreational MVPA (weekly total time in minutes spent in MVPA during recreational activities), using Global Physical Activity Questionnaire [GPAQ] instrument) [30], park time in minutes in the last month and park PA time in minutes in a typical month [20]. Based on formative research [31] we hypothesised five T1 survey measures as mediators of the outcomes at T2: motivation to engage in PA (based on Behavioural Regulation in Exercise Questionnaire- 2 questionnaire) [32], social support for PA, recreational MVPA minutes/week (from GPAQ), minutes spent in parks in the last month and minutes spent doing PA in parks in a typical month.

The rationale for the choice of the potential mediators of the interventions on the outcomes was as follows. To address barriers identified in the formative research, social support for PA in the form of group exercise was identified as a strategy, so we hypothesised social support for PA measured at T1 would be a mediator of outcomes at T2. Central to the logic for the intervention achieving outcomes is the idea that exposure to greenery via exercise in parks may have health benefits beyond exercise alone, and emphasising the restorative effects of nature during the counselling components of the intervention was identified as a strategy to address the barriers to PA. Therefore, park use and park PA measured at T1 were also hypothesised as mediators of the effect of intervention at T2. Since the intervention included several strategies to increase motivation to engage in PA generally, and the formative research identified goal setting and planning for PA to increase motivation specifically, motivation to engage in PA measured at T1 was also hypothesised as a mediator of outcomes achieved at T2. Mediators from T1 were chosen to allow us to evaluate the early impact of the ‘initial dose’ or treatment (before its full completion) as a possible mediator in the relationship between the intervention and the outcome. This was reasonable since four of the five intervention components were completed at T1.

### Process evaluation focus group methodology

For the process evaluation, the FGDs methodology was guided by phenomenological interpretive approaches [33, 34], which examined the experience of the park prescription trial from participants’ perspectives. Intervention group participants were purposefully sampled to join focus groups based on their distinctly high and low group exercise session participation rates. For the first focus group (FGD1), participants classified as regular participators (attended 68–100% of sessions) were approached during an exercise session as well as via text message and the first 12 who replied were included in the group. For the second (FGD2) and third FGDs (FGD3) a letter of invitation was sent to intervention group participants who were classified non-participators (attended 0% of sessions). FGD2 comprised nine non-participators and FGD3 comprised five non-participators and two participants who attended only three group exercise sessions. The people conducting the interviews had no involvement in the delivery of the intervention. The FGDs took place in a quiet room, with each discussion lasting 60–90 min. Participants were offered $20 to participate. The discussion was organised based on topic areas which mostly reflected the research questions of this process evaluation - (1) Perceived value of the program and its components, and (2) Barriers and facilitators to intervention participation. FGDs were conducted by a trained facilitator, whilst an observer took notes and managed the audio-recording. FGDs recorded amongst Mandarin speakers were transcribed to English (First and second FGD). Each section of the text was matched to a code representing each individual participant. Field notes were taken immediately after each discussion.

### Analysis

#### Describing implementation

Quantitative analysis of implementation includes descriptive statistics to summarise participation rates in each of the five elements of the intervention (i.e. dose) and the ratings for quality of and satisfaction with these elements.

#### Sub-group analysis to assess group exercise participation as a mechanism of impact

A comparison of the group exercise participation rates with the MVPA/week was made within the intervention group. The 71 intervention participants who completed the trial were grouped by the previously described (section 2.3.2) quantiles of exercise participation rates. Among the 71 participants, 62 provided complete accelerometer data for this analysis (*n* = 15, 13, 17 and 17 respectively for the four participation sub-groups). Linear regression compared accelerometer measured MVPA (minutes/week) at T2 among different participation groups, with and without adjusting for baseline self-report PA.

#### Mediation analysis to assess mechanisms of impact

Mediation analysis involved all intervention and control participants with complete survey data at T2. As a significant effect of the intervention on a mediator is commonly required to establish mediation [35–38], we first conducted simple linear regression analyses to evaluate the impacts of the intervention on the five hypothesised mediators, where the significant ones remained as potential mediators to assess their mediating effects. An alpha of 0.1 was used for exploratory purposes in this step instead of the default 0.05 elsewhere.

**Fig. 1:**
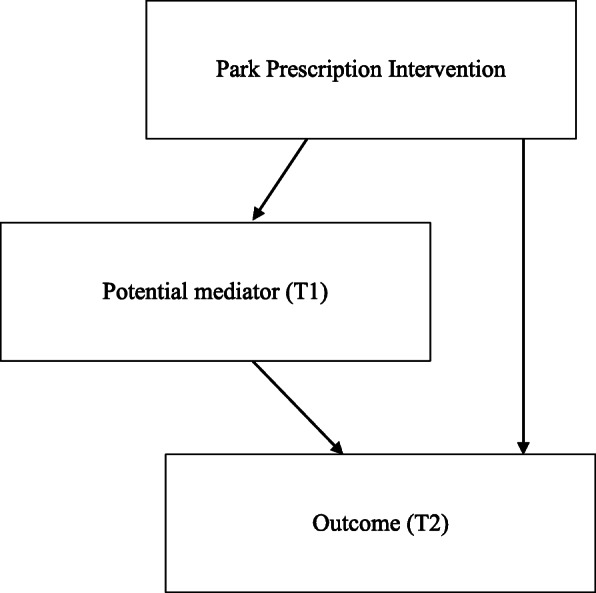
Path diagram of Structural equation modeling with a single potential mediator

To evaluate the effect of each potential mediator, we performed mediation analysis using structured equation modelling (SEM) for each of the four outcomes. Figure 1 illustrates the path diagram of single-mediator SEM: the nodes represent the variables included in the model, while the arrows indicate relationships between variables and the corresponding direction. Three types of effects of the intervention on the outcomes were quantified: indirect effects, direct effects and their sum - total effects. Indirect effects refers to the portions of total effects of the intervention that function through the mediator of interest, whereas direct effects account for the remaining part of the total effect [37]. Additionally, 95% CIs of the indirect effects were obtained from bootstrapping with 10,000 iterations. These SEMs were modelled using the R package ‘psych’ (version 1.7.2).

#### Qualitative analysis to explore implementation and mechanisms of impact

Framework analysis was conducted using a pre-determined framework of major themes reflecting the research questions [39]. The analysis used a more deductive approach since the research questions formed a pre-determined framework of themes, then sub-themes were derived at first by finding explicit meaning by staying close to the data, then by identifying codes reflecting more implicit meaning by relating this data to social and cultural phenomena as well as evidence from the literature. After familiarising themselves with the data, two analysts (N.P. and a Master of Public Health student who had completed a qualitative methods in public health unit) created codebooks before meeting to discuss them and agreeing on a near-final set of codes. These codes were sorted into themes, then the analysts reviewed the raw data under these themes to ensure coherence. For the mapping step of framework analysis, framework matrix charts containing data for each research question, with themes in columns and data beneath it divided in two rows of ‘participators’ (FG1) and ‘non-participators’ (FGD 2 and 3) were exported from NVivo (Pro version 12) by one analyst to individual worksheets in a spreadsheet and created by manually moving data into a spreadsheet by the other. This was done to explore similarities and differences in the discussion from participants with different rates of participation, which aligns with research question 3 of this process evaluation and complements the sub-group analysis (section 2.5.2). In the interpretation step, the two analysts reviewed the charts to make individual summaries of the findings. Following this, a third researcher (F. M.-R.) met with the analysts to discuss the summaries and agree on the findings.

## Results

### Study participants

PPI enrolled and randomised 80 participants into the intervention and control group, respectively, 145 (91%) of whom completed follow-up at 6 months. Table 2 shows participant characteristics. The participants were middle-aged (mean 51, SD ± 6.3 years) predominantly female (79.4%), of Chinese ethnicity (81.3%) and with an average Body Mass Index of 23.9 kg/m^2^ (SD ± 4.1 kg/m^2^), which reflects an increased risk of type 2 diabetes and cardiovascular disease among Asian populations [40]. There were three focus groups with 12 (FGD1), nine (FGD2) and seven (FGD3) participants respectively.

**Table 2:** Baseline participant demographic, behavioural, well-being, and health characteristics of the park prescription intervention participants (*N* = 80) and control group participants (N = 80)

Characteristics	Total (*N* = 160) n,%	PPI (N = 80) n,%	Control (N = 80) n,%
Age (mean, SD)	51.1 ± 6.3	52.1 ± 6.5	50.0 ± 6.0
Gender: Female	127 (79)	65 (81)	62 (78)
Ethnicity			
Chinese	130 (81)	67 (84)	63 (79)
Malay	14 (9)	7 (9)	7 (9)
Indian	13 (8)	5 (6)	8 (10)
Others	3 (2)	1 (1)	2 (2)
Education			
Secondary and below	84 (52)	41 (51)	43 (54)
Pre-tertiary	46 (29)	25 (31)	21 (26)
University and above	30 (19)	14 (18)	16 (20)
Work status: working	121 (76)	53 (66)	68 (85)
Marriage: Currently married	126 (79)	66 (82)	60 (75)
Household income/month (in Singapore Dollars)			
Below 2000	34 (21)	17 (21)	17 (21)
2000–3999	40 (25)	20 (25)	20 (25)
4000–5999	34 (21)	15 (19)	19 (24)
6000 and above	52 (32)	28 (35)	24 (30)
Physical activity related behaviours			
Total MVPA, minutes/week^a^ (mean, SD)	442.7 ± 534.7	475.7 ± 618.1	409.8 ± 437.2
Time spent in Park, minuets/month (mean, SD)	171.4 ± 293.8	168.1 ± 303.2	174.7 ± 286.1
PA in Park, minuets/month (mean, SD)	130.3 ± 261.8	132.7 ± 296.6	127.9 ± 223.6
Mental well-being			
WHO5 total, range: 0–100 (mean, SD)	58.3 ± 22.3	58.1 ± 22.1	58.5 ± 22.6
Physical Health			
BMI, kg/m2 (mean, SD)	23.9 ± 4.1	24.2 ± 4.1	23.6 ± 4.1

Note: Data are mean ± SD or n (%) unless otherwise indicated

^a^Subjective measures based on GPAQ

### Dose of each intervention component received by participants during implementation

**Fig. 2:**
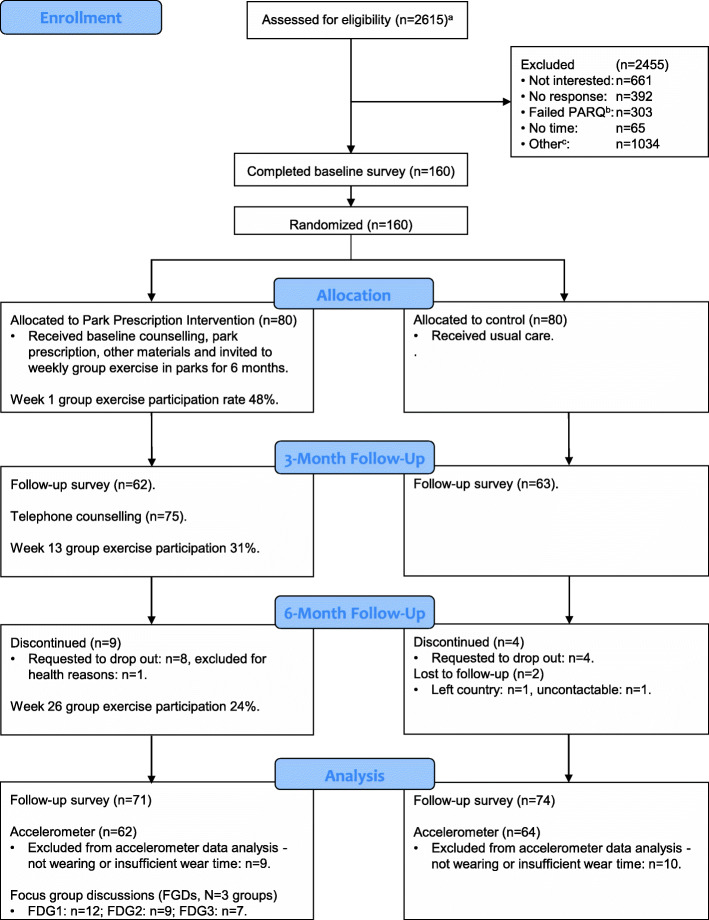
Flow of participants through intervention components, follow-up measures and analysis. **a** Exact number of participants screened could not be determined due to the nature of recruitment from multiple community screenings. **b** PARQ: Physical Activity Readiness Questionnaire. **c** Invalid phone number, technical communication issues, non-English speakers, illiterate, moved to different region in Singapore

Figure 2 shows the flow of participants through the study. All intervention group participants received the baseline counselling, park prescription and materials at the commencement of the program. Five participants did not participate in follow-up counselling at three-months. Of these, three could not be contacted after more than four attempts on separate days, one refused follow-up counselling and another withdrew from the study at three-month follow-up. Group exercise participation rates varied amongst the intervention group as illustrated in the quantiles of participation in section 2.3.2. Overall intervention group exercise participation rates declined from 48% at baseline to 31% at three-months and 24% at 6 months PPI completion.

### Quantitative results for satisfaction with the intervention during implementation

Survey results from intervention participants on the quality of and satisfaction with each component of the intervention are summarised here and shown in detail in the Supplementary File Table S1 (Editors, please create hyperlink). At T1 the quality of the park prescription was and the satisfaction with materials were assessed, whereas at T2 the quality of the follow-up counselling and the program overall evaluated. For T1 questions about quality of the prescriber’s delivery of the park prescription most (83–93%) of participants agreed/strongly agreed with a set of questions which indicate the prescriber delivered a quality park prescription. Overall, 68% were moderately/very satisfied with the prescriber’s delivery of the prescription and 32% were slightly/somewhat satisfied. Responses to the quality of the materials were mixed - 75% felt they were easy to understand and trustworthy, but responses were split in relation to how much participants liked them (slightly/somewhat: 51%, moderately/very: 49%). In contrast, at T2 a high proportion of participants (82–94%) agreed/strongly agreed to questions supporting the quality of the follow-up counselling and 79% were very/extremely satisfied with the counselling. Seventy-six percent were very/extremely satisfied with the intervention overall.

### Sub-group analyses to assess the impact of group exercise participation on MVPA

Mean MVPA/week (95% CI) according to the levels of group exercise participation differed significantly (*p* = 0.018): non- participators 128.3 (69.3, 187.2) minutes, irregular participators 100.3 (36.9, 163.6) minutes, semi-regular participators 50.5 (− 4.9, 105.9) minutes and regular- participators 177.4 (122.0, 232.8) minutes. The difference remained significant after adjusting for baseline self-report MVPA (*p* = 0.024).

### Mediation analysis to assess mechanisms of impact

Three of the five hypothesised mediators measured at T1 were affected by the intervention and therefore selected as potential mediators: motivation (effect size: 5.8, 95% CI -1.1, 12.7, p: 0.100), recreational PA (effect size: 122.3 min, 95% CI 70.1, 393.9, p: 0.092) and park PA time (effect size: 232.0 min, 95% CI -20.2, 264.7, p: 0.005).

Table 3 shows results of SEM. For the park PA time and motivation mediators measured at T1, 120 participants provided survey data, whilst 119 provided data for the park time. A total of 145 participants provided survey data for the T2 intervention outcome effects. Park PA time at three-months had a significant mediating effect (95% CI) on all investigated outcomes: recreational PA per week 26.50 (6.65, 49.37) minutes, time spent in parks 185.38 (45.40, 353.74) minutes/month, PA time in parks in a typical month 165.48 (33.14, 334.16) minutes and psychological quality of life score 1.25 (0.19, 2.69). In contrast, motivation and recreational PA did not demonstrate a statistically significant mediation effect.

**Table 3:** Intervention total, direct and indirect effects on the four outcomes from mediation analysis

Outcome at 6 months (***N*** = 145)^**b**^	Potential Mediator (***N*** = 120)^**c**^	Total Effect	Direct Effect	Indirect Effect
		estimate (CI)	estimate (CI)	estimate (95% CI from bootstrapping)
Recreational MVPA (minutes/week)	Motivation (T1)	46.19 (0.46, 91.92)	41.68 (− 4.46, 87.82)	4.19 (− 2.63, 14.06)
	Recreational MVPA (T1)		42.39 (− 3.88, 88.67)	4.64 (−4.41, 19.87)
	Park physical activity time (T1)^**a**^		19.37 (−24.77, 63.52)	26.50 (6.65, 49.37)^**a**^
Park time in last month (minute/month)	Motivation (T1)	151.92 (11.42, 292.42)	131.79 (−9.25, 272.83)	18.86 (−5.37, 54.08)
	Recreational MVPA (T1)		113.12 (−23.67, 249.91)	47.63 (−4.41, 165.45)
	Park physical activity time (T1)^**a**^		−37.06 (−129.25, 55.12)	185.38 (45.40, 353.74)^**a**^
Park physical activity time (minutes/month)	Motivation (T1)	190.29 (60.16, 320.42)	177.55 (46.21, 308.90)	13.02 (−3.38, 40.94)
	Recreational MVPA (T1)		156.84 (29.65, 284.03)	42.79 (−6.47, 154.37)
	Park physical activity time (T1)^**a**^		23.68 (−62.79, 110.15)	165.48 (33.14, 334.16)^**a**^
Psychological quality of life	Motivation (T1)	4.05 (0.04, 8.05)	3.43 (−0.58, 7.43)	0.56 (−0.22, 1.67)
	Recreational MVPA (T1)		3.63 (−0.41, 7.68)	0.43 (−0.06, 1.01)
	Park physical activity time (T1)^**a**^		2.79 (−1.28, 6.85)	1.25 (0.19, 2.69)^**a**^

^**a**^Significant mediating effect

^**b**^N of outcomes, 144 for Park time in last month (T2) and 145 for the rest

^**c**^N of mediators: Motivation (T1) 120; Recreational MVPA (T1) 119; Park physical activity time (T1) 120

### Qualitative exploration of implementation and mechanisms of impact

Table 4 summarises the participants’ views according to research questions. The intervention was valued by participators and non-participators alike and there was a strong preference by all participants for the interactive components of the program (e.g. counselling and SMS reminders), which resulted in the theme ‘a little less paper, more interaction’. Discussion pertaining to barriers and facilitators to participation in the intervention was categorised into dispositional (i.e. relating to participants own subjective disposition) and situational (i.e. relating to participants fixed, objective situation) themes. There was a larger volume of discussion under barriers to participation amongst group exercise non-participators. When exploring explanations beyond a lack of time to engage with the intervention, a lot of the comments were dispositional, where participants were making a choice to prioritise whatever time is left from a busy week for family or domestic work. There were differences in participators’ and non-participators’ comments on this topic - a preference for unstructured PA was only discussed by non-participators. Discussion on facilitators to engage in the intervention was grouped into two main themes of external and internal enabling factors. External motivators included social interaction and the group dynamic, the knowledge and skills of the group exercise instructor and the follow-up. Comments on the group dynamic being a motivator were made exclusively by those who attended the group exercise, but discussion on prompts and follow-up being a motivator came from both participators and non-participators. Sub-themes for internal motivating factors included developing a routine to attend sessions, perceived health susceptibility, empowerment and other individual factors. Developing a routine to attend exercise sessions, allowing them to build it into their weekly schedules and other factors such as managing one’s personal health were discussed mostly by group exercise participators. Perceived health susceptibility and the theme of being empowered by participation in the intervention or through their own exercise were discussed by participators and non-participators.

**Table 4:** Focus group discussions themes, elaboration, illustrative quotes and potential implications beneath research questions^a^

Themes (Sub-themes)	Elaboration of themes/sub-themes	Illustrative “Quotes” and points of discussion
**Research question 1: To what extent do participants value the program overall and which components of the program do participants perceive as valuable?**		
Participants appreciate the program.	Non-attenders and attenders all appreciated the program. Attenders wanted the program to be sustained. They described being empowered to continue regardless of the program itself continuing, which may relate to their connection with the group.	*“The most I just do a bit of walking to the market that’s all. So after, this program started,* ***I make more regular trips to the park*** *(FGD3, P5).”*
The exercise sessions were valued by attenders. Telephone follow-up was valued by all.	The exercise sessions were valued by attenders. The phone counselling was described as a motivator by attenders and non-attenders.	Program re-design could consider that non-attenders found follow-up a motivator for unstructured activity.
The park prescription was referred to initially.	It was referred to initially, but they felt they knew it and did not need to refer back to it.	One participant suggested the prescription could be repeated.
The planning sheet was used more as a recording tool, mostly by attenders.	This was consistent across attenders and non-attenders. A common explanation is that the activities they engage in don’t vary much.	Some suggestions were provided for condensing it. Future programs may need to encourage completing it.
Less paper, more interaction	All groups commented positively about reminders including telephone follow-up.	WhatsApp messages from people in their exercise classes were spontaneous.
**Research question 2: What do participants describe as barriers to intervention participation? (Beyond time, what are their justifications for non-participation?)**		
Situational and dispositional justifications for not attending the exercise classes: No time! Family and work come first.	Non-attenders provided most of these justifications. Family time and work commitments were common justifications. Some discussion was situational (e.g. clashes with work), some was dispositional (e.g. choosing to prioritise time for family and domestic work).	An example of a barrier to participation only mentioned by the attenders was a lack of motivation.
Preference for unstructured activity in their own time.	Another time-related justification discussed only by non-attenders was a preference for unstructured activities that they could do in their own time.	“For me, *after work I* ***walk to MRT*** *.. my office is on the 6th floor; I will* ***walk up*** *every morning.” (FGD3P1)*
**Research question 2 (continued): What do participants describe as facilitators to intervention participation? External and Internal motivators**		
External: Socialisation and the group dynamic.	There was a large volume of discussion about this as a motivator for attendance by attenders. There was an obvious bond amongst the participants, who shared how exercising is fun because of the group.	*“I’m not those really disciplined type. But I feel that I need more kakis, so that they’ll encourage...” (FGD3, P6) [Kaki’s is a colloquial term for ‘buddy’ or ‘one of us’].*
External: Knowledge and skills of the instructor.	This was a strong theme amongst attenders. Passing on technical information, tailoring exercises to the individual and building rapport with attendees were mentioned.	*“..he’ll tell us that this will help do this... In fact it’ll* ***add to our additional knowledge****, or our daily life.” (FGD3, P4).*
External: Being monitored and reminded.	Contact via the telephone counsellor was highly valued. This was discussed by all groups.	“*Like* ***a wake up, woah you call****, better buck [up].” (FGD3, P7)*
Internal: Developing a routine to attend sessions.	Developing a routine meant people attended these sessions as part of their everyday lives.	*“Don’t know leh; I’m like ‘time to [exercise],* ***don’t go out, will feel very uncomfortable.”*** *(FGD1, PX).*
Internal: Perceived health susceptibility and perceived benefits to health.	Health as an internal motivator to program adherence was a strong theme across all groups, and this discussion often related to their age. They also noted the perceived benefits of attending the sessions such as having more energy, better mood and improved physical functioning.	“*For me it’s uhh, I’ve* ***high cholesterol*** *[…*] *So got a bit of improvement lah.” (FGD2, P7).* *“Ah, body system will be healthier, .. have* ***more energy****, and will be beneficial to* ***mood*** *as well.” (FGD1, P8)*
Internal: Empowerment.	Being empowered, either by the program to do group exercise just by doing their own exercise, was implicit in comments across all groups.	*“I just do a bit of walking to the market .. So after, this program .., I* ***make ..regular trips to park*** *(FGD3P5).”*
Internal: Other individual factors.	A variety of factors were also mentioned as motivation for program participation, mostly in the context of attending the exercise sessions. Having personal responsibility for one’s health, for financial savings in terms of the cost of maintaining health were examples.	“*In Singapore, you can die but you cannot fall sick. You won’t have the ability. You have to...* ***rely on yourself”*** *(FGD2, P4).*

^a^Comparing all FGD amongst group-exercise participators (FGD 1) and non-participators (FGDs 2, 3) contributed to addressing research question 3 of this process evaluation

## Discussion

The Park Prescription intervention effectively increased recreational PA, park use, park PA, and psychological quality of life. Although intervention participants achieved greater accelerometer measured MPVA time (minutes/week) than controls, this result was not statistically significant [20]. The implementation and the mechanisms of impact of the intervention have been explored in this process evaluation and this discussion integrates quantitative and qualitative findings to address the four research questions.

### Did participants value the intervention and its components?

Results from the quantitative survey questions and the FGD prompts relating to whether participants valued the program and each component were consistent. Most participants valued the program highly and the FGDs found that this was common to both participators and non-participators of group exercise sessions, which may indicate that for non-participators components of the program other than group exercise were valued. The quote from a non-participator in group exercise reflects this: *“The most I just do a bit of walking to the market, that’s all. So after this program started, I make more regular trips to the park (FGD3, P5).”* Quantitative and qualitative findings suggested that the paper-based components of the program were less valued than the interactive components, which is discussed further in relation to other research questions below.

### What did participants perceive as barriers and facilitators to intervention participation?

Results for implementation dose showed that almost all participants received four of the five intervention components; the exception being the group exercise sessions. This led to the FGDs exploring justifications for non-participation in the group exercise amongst distinct groups of participators and non-participators. The themes formed two main categories – dispositional and situational. The dispositional barriers were common to all groups and formed a large portion of discussion (particularly amongst non-participators in group exercise). Intervention group participants were discussing choosing to spend what little time they had left in a busy working week with family or for domestic duties. These participants were mostly middle-aged women of Chinese ethnicity who worked. In many Chinese societies there is a strong feeling of duty to family, also termed filial piety [41], which could be considered in delivering interventions to this population through involving partners or other family members to also participate, or to encourage the family member to make time for themselves to participate. There is evidence of effectiveness of recruiting adult family dyads to healthy lifestyle interventions [42] which could inform such adaptations of the present intervention and interventions like it.

Facilitators for intervention participation discussed by FGD participants were grouped into extrinsic and intrinsic factors. Interestingly, some of the factors described in both the participator and the non-participator FGDs are factors which increase the feasibility of delivering effective interventions at scale [43] since they are relatively low intensity and inexpensive. For example, in all the FGDs many participants expressed valuing various forms of follow-up. This included the telephone follow-up that was part of the program and forms of follow-up that the participants spontaneously established, like using app-based messaging services (WhatsApp) to prompt participation. A participant quote illustrated this: “*Halfway my child finished his exam, … WhatsApp’ed ‘come ah come ah’, I then attended again.” (FGD1, PX).* A recent review identified that m-Health strategies show promise for improving the effectiveness of interventions [44] and a study from China which compared follow-up via telephone or via app-based messaging (WeChat) found that both achieved similarly low rates of loss to follow-up, but app-based follow-up took significantly less time [45]. Perceived health susceptibility was also mentioned as a common internal motivator for intervention participation, which may have implications for how interventions targeting these populations communicate with participants. Another common internal motivating factor to be physically active was having personal responsibility for one’s health, for financial savings related to the cost of maintaining health, as mentioned by a participant in the second focus group: “*In Singapore, you can die but you cannot fall sick. You won’t have the ability. You have to... rely on yourself” (FGD2, P4).* This issue is particularly relevant in some Asian countries where universal health coverage does not yet exist, or there may be significant gaps between the cost of health services and the government funded component and the cost of health care may be high relative to incomes [46, 47].

### Did a higher level of group exercise participation correspond with greater PA per week?

Sub-group analysis revealed that although MVPA was significantly different between the different levels of group exercise participation the pattern was not linear. Non-participators achieved greater MVPA than irregular participators and semi-regular participators, whilst only regular participators achieved over 150 min of MVPA/week recommended for maintaining health in international PA guidelines [48]. Whilst exploring barriers and facilitators to participation, an FGD sub-theme arose illustrating that some non-participators in group exercise preferred unstructured activity. They discussed being motivated by the intervention to do this in their own time. The formative research conducted to develop the PPI also found, that the target population of the intervention appeared to have a preference for shorter and less intense exercise [31]. A population survey of 1332 adults (response rate 46%) selected randomly from an Australian electoral role, which included questions on participants’ preferences for structured versus unstructured PA found that 53% reported enjoyment of unstructured PA and 31% reported enjoyment of structured PA. However, those reporting enjoyment of structured PA were twice as likely to report sufficient participation in vigorous activities [49]. The findings of the Australian cross-sectional study and our prospective study suggest that in any population there will be mixed preferences for structured versus unstructured PA, and for those that enjoy unstructured PA attention may need to be given to encouraging them to gradually increase the intensity and duration of this activity. Therefore, intervention effectiveness could be enhanced by tailoring unstructured options for park PA for participants who indicate a preference for it. It may also be necessary to encourage gradual increases in duration and intensity of unstructured exercise so that PA goals are achieved.

### Did hypothesised factors mediate the effect of the intervention on the outcomes?

This study illustrates that park PA at three-month follow-up was a consistent and strong mediator for the observed effects of the intervention on recreational PA, psychological quality of life, park use and park PA at six-month follow-up. Reviews of the evidence of a relationship of parks and green space with health [14, 15, 17] have highlighted that few studies have aimed to understand the mechanisms by which parks and green space can improve health, and available studies exploring mechanisms have only used cross-sectional data [13, 19, 50, 51]. This study used the results from an RCT to explore potential mediators and thereby adds to these previous studies. Since the measurement of mediators preceded the outcomes this suggests a temporal relationship by which an intervention promoting exposure to nature and PA achieved behavioural and health outcomes. The finding that PA in public parks (also called green PA) mediated the intervention effects whilst other hypothesised mediators of recreational PA and park time alone did not demonstrate a mediating effect warrants further attention. Similar to our study, de Vries and colleagues explored three potential mediators (stress reduction, stimulating PA and facilitating social cohesion) of the relationship between exposure to greenery and health [13]. They found that although total PA was not a mediator, PA in public green space was. Their cross-sectional study is not directly comparable to the present study for reasons including the exposure being street side greenery near the participants’ homes and the outcome measure being a question on general health from the Short-Form 36 questionnaire. However, the somewhat similar findings of both studies merit future research to assess how green PA is a mechanism by which interventions can improve health.

### Strengths and limitations of the process evaluation

A strength of this process evaluation is that it combines qualitative and quantitative data to explore the mechanisms of impact of the intervention in depth. The data from the RCT is prospective, with six-months of follow-up, therefore it infers a temporal relationship between the intervention, mediating factors and the outcomes achieved. The process of randomisation to intervention and control groups also balances potential moderating factors and possible confounding factors between the two groups to provide more confidence in the identified mediation effects. The impacts of and reasons for the varied levels of participation in group exercise participation were also explored in great detail, first by conducting sub-group analysis to explore quantitative impacts on the main study outcome, then by using framework analysis to explore similarities and differences for barriers and facilitators to participation using qualitative data. This provided plausible reasons for the observed impacts of varying levels of group exercise participation on the main study’s outcomes.

The process evaluation also had some limitations. Information on fidelity of implementation was not included since there were no formal records of observations of intervention delivery. However, study staff were trained using protocols for consistent delivery of each component of the intervention, group exercise sessions were observed regularly by the study team member leading the implementation and anecdotal feedback on whether the intervention was delivered as per the protocols was obtained from the staff who implemented the intervention. Furthermore, the focus of this process evaluation was to explore the intervention’s mechanisms of impact, which is appropriate for a process evaluation of an effectiveness study [22]. Since focus group participants were only recruited from the 80 participants in the intervention arm of the trial, there was a risk that data saturation (i.e. information being repeated to a point where no new information emerges from the data) may not have been achieved. However, the concept of data saturation is not always useful in thematic analysis since it has been argued that it is always possible new information may arise [52], for most of the research questions it was only appropriate to engage these participants, they provided valuable insights and some of these were consistent with FGD feedback from the formative research that was conducted to develop the intervention [31]. Mediation analysis tested the effects of mediators at 3 months on the 6 month outcomes to evaluate the early impact of the ‘initial dose’ or treatment as a possible mediator, yet some participants continued to receive the group exercise component up to 6 month. However, participation rate for the weekly exercise was 31% at 3 months, and this declined further to 24% at 6 months, so the effect of dose-response may not have greatly impacted the results of the mediation analysis. To further support this postulation, separate analyses were conducted using the same mediators at 6 months and the results did not change substantially. The population was middle-aged and predominantly Asian, which may limit the generalisability of some findings to other populations.

## Conclusions and recommendations

The Park Prescription Intervention showed promise for promoting PA, increasing park use and improving wellbeing in community settings. This process evaluation explored the mechanisms of impact of the intervention and identified that park PA consistently mediated effects of the park prescription intervention. This suggests being active in nature may be an important mechanism of the intervention effects on behavioural and quality of life outcomes. The varying levels of participation in the group exercise component of the intervention may be explained by qualitative feedback from non-participators noting that they preferred unstructured PA and the intervention motivated them to do this. To optimise intervention effectiveness, key barriers to intervention participation which may have contributed to not achieving some health outcomes may need to be addressed. First, tailoring the intervention to participants’ preferences for unstructured or structured PA, with an emphasis on encouraging those who prefer unstructured PA to gradually increase the volume and intensity. Second, considering participants’ strong preference for prioritising time with family instead of PA by inviting family-members to the intervention or by engaging them to provide support and encouragement.

## Supplementary Information

**Additional file 1.**

## Data Availability

The dataset(s) supporting the conclusions of this article are available upon reasonable request to the corresponding author and after the National Parks Board of Singapore approves its provision.

## Abbreviations

PA: Physical activity
MVPA: Moderate-to-vigorous physical activity
FGD: Focus group discussion
SEM: Structural equation modelling
PPI: Park prescription intervention
CI: Confidence interval
N, n: Number of participants
U.S.: United States of America
QoL: Quality of life
RCT: Randomized controlled trial
I: Intervention group
C: Control group
T0: Baseline
T1: Three month follow-up
T2: Six-month follow-up
WHO: World Health Organisation
SD: Standard deviation
Kg: Kilogram
m^2^: meter squared
GPAQ: Global Physical Activity Questionnaire
IPAQ: International Physical Activity Questionnaire
PARQ: Physical Activity Readiness Questionnaire
P: Participant
