# Supplementary material for: Activity in nature mediates a park prescription intervention’s effects on physical activity, park use and quality of life: a mixed-methods process evaluation
Source: BMC Public Health. 2021 Jan 22;21:204. doi: 10.1186/s12889-021-10177-1 (PMC7825241; doi:10.1186/s12889-021-10177-1)
Supplement: Supplementary file 1 — Additional file 1. [file 12889_2021_10177_MOESM1_ESM.docx]

Table S1. Participant feedback on the quality of and satisfaction with intervention components

| Intervention component and related question | | Response, n (%) | | | |
| --- | --- | --- | --- | --- | --- |
| Prescriber (three month questionnaire) | **Disagree/**  **Strongly disagree** | | **Neither disagree nor agree** | **Agree/**  **strongly agree** | |
| Explained the reason(s) for prescription? | 1(2) | | 7(12) | 51(86) | |
| Easy to understand? | 0(0) | | 6(10) | 53(90) | |
| Confident of prescriber’s knowledge and skills? | 0(0) | | 5(8) | 54(92) | |
| Showed respect for what I had to say? | 0(0) | | 4(7) | 55(93) | |
| Listened carefully? | 0(0) | | 5(8) | 54(92) | |
| Encouraged me to talk about my concerns? | 0(0) | | 8(14) | 51(86) | |
| Spent enough time with me? | 0(0) | | 10(17) | 49(83) | |
| Prescriber overall satisfaction | **Not at all** | | **Slightly/**  **Somewhat** | **Moderately/Very** | |
| Overall experience with the prescriber? | 0(0) | | 19(32) | 40(68) | |
| Materials (prescription sheet and park brochure at 3 months) | **Not at all** | | **Slightly/**  **Somewhat** | **Moderate/Very** | |
| How attractive are they? | 0(0) | | 25(42) | 34(58) | |
| How informative are they? | 0(0) | | 19(32) | 40(68) | |
| How easy to understand are they? | 0(0) | | 15(25) | 44(75) | |
| How encouraging are they? | 1(2) | | 26(44) | 32(54) | |
| How useful in helping you be more physically active are they? | 1(2) | | 22(37) | 36(61) | |
| How much do you like them? | 0(0) | | 30(51) | 29(49) | |
| How trustworthy are they? | 0(0) | | 15(25) | 44(75) | |
| Were you already familiar with the information in them? | 4(7) | | 35(59) | 20(34) | |
| How likely are you to make lifestyle changes based on them? | 0(0) | | 34(58) | 25(42) | |
| Planning sheet | **Not at all** | | **Slightly/**  **Somewhat** | **Moderately/Very** | |
| How attractive is it? | 2(3) | | 30(52) | 26(45) | |
| How informative is it? | 1(2) | | 22(38) | 35(60) | |
| How easy is it for you to understand? | 1(2) | | 21(37) | 35(61) | |
| How encouraging is it? | 3(5) | | 22(38) | 33(57) | |
| How useful is it in helping you to be more physically active? | 3(5) | | 20(34) | 35(60) | |
| How much do you like it? | 2(3) | | 27(47) | 29(50) | |
| How trustworthy is it? | 2(4) | | 24(42) | 31(54) | |
| Were you already familiar with the information in it? | 6(10) | | 28(48) | 24(41) | |
| How likely are you to make lifestyle changes based on it? | 3(5) | | 26(45) | 29(56) | |
| Follow-up counselling (six month questionnaire) | **Disagree/**  **Strongly disagree** | | **Neither disagree nor agree** | **Agree/**  **Strongly agree** | |
| Explained the reason(s) for giving me the phone counselling | 1 (2) | | 11 (17) | 54 (82) | |
| Explained things in a way that was easy for me to understand | 0 (0) | | 4 (6) | 62 (94) | |
| I was confident of the counsellors knowledge and skills | 0 (0) | | 6 (9) | 60 (91) | |
| Showed respect for what I had to say | 1 (2) | | 4 (6) | 61 (92) | |
| Listened carefully to me | 1 (2) | | 3 (5) | 61 (94) | |
| Encouraged me to talk about all my concerns | 2 (3) | | 5 (8) | 59 (89) | |
| Spent enough time with me | 1 (2) | | 11 (17) | 54 (82) | |
| Follow-up counselling overall satisfaction | **Not at all** | | **Slightly/**  **Moderately** | **Very/Extremely** | |
| How would you rate your overall experience? | 0 (0) | | 14 (21) | 52 (79) | |
| Project overall satisfaction (six month questionnaire) | **Not at all** | | **Slightly/**  **Moderately** | **Very/Extremely** | |
| How would you rate your overall experience? | 0 (0) | | 17 (24) | 53 (76) |  |

Where n does not equal 62 at three months and 71 at six months there are missing responses. Some row percentages may not add to 100 due to rounding.
